# Acute Beetroot Juice Supplementation Attenuates Morning-Associated Decrements in Supramaximal Exercise Performance in Trained Sprinters

**DOI:** 10.3390/ijerph18020412

**Published:** 2021-01-07

**Authors:** Amanda M. Dumar, Anna F. Huntington, Rebecca R. Rogers, Thomas J. Kopec, Tyler D. Williams, Christopher G. Ballmann

**Affiliations:** Department of Kinesiology, Samford University, 800 Lakeshore Dr., Birmingham, AL 35229, USA; adumar@samford.edu (A.M.D.); ahunting@samford.edu (A.F.H.); rrogers1@samford.edu (R.R.R.); tkopec@samford.edu (T.J.K.); twilli11@samford.edu (T.D.W.)

**Keywords:** nitrite, wingate, sprint, anaerobic capacity

## Abstract

Diurnal fluctuations in power output have been well established with power loss typically occurring in morning (AM) times. Beetroot juice (BRJ) is a source of dietary nitrate that possess ergogenic properties, but it is unknown if ingestion can mitigate performance decrements in the morning. The purpose of this study was to examine the effects of acute BRJ supplementation on diurnal fluctuations in anaerobic performance in trained sprinters. Male Division 1 National Collegiate Athletic Association (NCAA) sprinters (*n* = 10) participated. In a double-blinded crossover study design, participants completed three counterbalanced exercise trials under different conditions: Morning–placebo (8:00 HR, AM-PL), Morning–BRJ (8:00 HR, AM-BRJ), and Afternoon–no supplement (15:00 HR, PM). For each trial, participants completed 3 × 15 s Wingate anaerobic tests separated by 2 min of rest. Each trial was separated by a 72 h washout period. Mean power output (*p* = 0.043), anaerobic capacity (*p* = 0.023), and total work (*p* = 0.026) were significantly lower with the AM-PL condition compared to PM. However, BRJ supplementation prevented AM losses of mean power output (*p* = 0.994), anaerobic capacity (*p* = 0.941), and total work (*p* = 0.933) in the AM-BRJ compared to the PM condition. Rate of perceived exertion was not significantly different between any conditions (*p* = 0.516). Heart rate was significantly lower during the AM-BRJ condition compared to AM-PL (*p* = 0.030) and PM (*p* < 0.001). Findings suggest anaerobic capacity suffers during AM versus PM times in trained sprinters, but BRJ ingestion abolishes AM-associated decrements in performance.

## 1. Introduction

Circadian rhythms are psychological, physiological, and physical oscillations that follow a 24 h pattern [1,2,3]. Although circadian rhythms are endogenously self-sustained, they may be influenced by various external factors including light–dark cycle, environment, ambient temperature, and feeding state/dietary intake [4,5]. Although some disparities exist, physical performance has been repeatedly shown to have diurnal fluctuations with peak performance typically associated with afternoon (PM) times versus morning (AM) [6,7]. Because of performance decrements during AM times, athletes and competitors commonly use behavioral and dietary modifications to optimize training and performance during various times of day [8]. These interventions may potentiate performance during peak performance times or prevent the loss of performance during non-optimal times of day [9,10].

Time-of-day dependent changes in exercise performance and power output have been well described, albeit some conflicting evidence exists [6,9,11,12,13]. Chtourou et al. showed higher power output and performance in short-term supramaximal cycling performance during PM versus AM times [11]. Higher performance levels were concomitant with improved neuromuscular efficiency and mean power frequency. Zarrouk et al. reported that peak power and total work during repeated sprints suffered in AM versus PM times [12]. Power and barbell velocity during explosive resistance exercise have also been reported to be lower during AM times by multiple investigations [6,9,10,14]. While mechanisms for changes in performance due to time of day are not fully understood, most evidence suggests underlying changes in core body temperature, muscular ionic movement, and substrate utilization [3]. However, others have shown limited changes in performance with time of day. Deschenes et al. showed no changes in exercise performance across various times of the day although variations in sympathoadrenal activity existed [15]. Lack of differences in anaerobic exercise recovery in AM versus PM times have similarly been shown [16]. Interestingly, Lericollais et al. showed lower power output during AM times, but fatigue was unaffected in elite trained cyclists [13]. These conflicts suggest the need for further study on diurnal fluctuations and exercise performance especially in well-trained athletes.

Beetroot juice (BRJ) is an abundant source of inorganic dietary NO_3_^−^ [17]. Once ingested, the NO_3_^−^ is reduced by oral microbiota into nitrite (NO_2_^−^), which then increases in the systemic circulation [18]. The circulating NO_2_^−^ then gives rise to nitric oxide (NO^−^) that has been shown to increase vasodilation, improve blood flow, increase ATP synthesis, and increase lactate clearance [19,20]. A myriad of evidence exists supporting ergogenic benefits of BRJ supplementation [20,21,22,23]. Wylie et al. reported increases in power output during repeated short sprints with 5 days of BRJ intake [20]. Furthermore, Coggan showed increased contraction velocity during knee extensor exercise with acute BRJ ingestion [22]. Others have similarly shown improvements in explosive resistance exercise performance with BRJ supplementation [21,23]. Recently, Williams et al. reported increases in explosive bench press power and barbell velocity with acute BRJ ingestion [21]. Improvements in power output and exercise performance may be due to increased neuromuscular efficiency, reduced ATP cost for contraction, and increases in calcium sensitivity [20,24]. To date, less is known on whether dietary enrichment with BRJ influences AM dependent changes to exercise responses. 

Given that the ability to develop power during intense exercise appears to be altered by the time of day in which the exercise is performed, most previous investigations on BRJ supplementation strictly control for the time in which exercise sessions are performed [21,22,23,25]. Although there is little variation in endogenous NO_2_^−^ production from day to day, NO_2_^−^ levels appear to be lowest during early morning hours [26]. Furthermore, phosphocreatine (ATP-PC) and glycolytic flux have been suggested to be lower during AM times possibly due to fluctuations in core body temperature, which may negatively impact repeated exercise performance [27]. Taken together, exogenous NO_3_^−^ ingestion in the morning through BRJ may aid to increase lower AM NO_2_^−^ levels, thereby improving skeletal muscle blood flow, ATP-PC resynthesis, and attenuate AM-dependent losses in power output. While previous investigations have shown NO_3_^−^ supplementation improves performance when times of day is standardized, no studies to date have determined if NO_3_^−^ supplementation can prevent diurnal changes in anaerobic performance. Thus, the purpose of the following study was to investigate if acute BRJ supplementation could attenuate AM-associated losses in anaerobic performance during supramaximal anaerobic sprint performance in trained sprinters. We hypothesized that exercise performance would suffer during AM versus PM times but that BRJ ingestion would attenuate AM decrements in anaerobic capacity and performance and result in similar to peak performance times in the PM.

## 2. Materials and Methods

### 2.1. Study Design

The following investigation used a double-blinded, counterbalanced, crossover study design to investigate the effects of acute BRJ ingestion on supramaximal WAnT sprint performance. Division I National Collegiate Athletic Association (NCAA) male sprinters completed three randomized exercise trials: (1) Morning (8:00 HR) with placebo (AM-PL), (2) Morning (8:00 HR) with BRJ (AM-BRJ), and (3) Afternoon (15:00 HR) with no supplementation. For each trial, participants completed 3 × 15 second Wingate anaerobic tests (WAnT) separated by 2 min of rest. All trials were separated by a 72 h washout period.

### 2.2. Participants

An a priori power analysis was used to determine adequate sample size using G-power 3.1.9.6 software. A previous investigation on acute BRJ ingestion and power output during resistance exercise performance showed estimated effect sizes for power output improvements at f = 0.36 and correlation of repeated measures = 0.91 [21]. Accordingly, the following parameters were used: test = repeated measures ANOVA, f = 0.36, α = 0.05, β = 0.8, correlation among repeated measures = 0.91. This yielded a minimum sample size of *n* = 6. To be consistent with participant numbers of previous investigations on BRJ and exercise performance, a sample size of *n* = 10 was selected [20,21,28]. Thus, ten male Division I NCAA sprinters (age = 20.3 yrs ± 1.9, height = 182.5 cm ± 5.2, body mass = 76.1 kg ± 6.1) were recruited for this study. All the sprinters were on the same collegiate team and followed similar practice and training protocols. To participate, individuals had to be currently active on a Division I collegiate track and field roster. Participants were excluded if they had a history of lower body injuries within 6 months prior to the initial testing date or allergies to beetroot juice or any of its constituents. Screening for exercise safety was completed using a physical activity readiness questionnaire (PAR-Q). All individuals refrained from vigorous lower body exercise 24 h prior to each trial. Additionally, participants were asked to refrain from consuming caffeine, nicotine, preworkout supplements, alcohol, and antibacterial mouthwash at least 12 h prior to testing. Written and informed consent was obtained from each participant prior to data collection. All experimental procedures were conducted in accordance with the Declaration of Helsinki and approved by the Samford University Institutional Review Board (EXPD-HP-20-SUM-17). 

### 2.3. Supplementation

For each AM trial, participants ingested a single dose of 70 mL of concentrated BRJ (Sport shot, Beet It, United Kingdom) or PL (black currant juice; Suntory, Tokyo, Japan) 2 h before each exercise trial [21,29]. The NO_3_^−^ content of the BRJ was standardized to provide approximately 400 mg while the PL provided negligible to no NO_3_^−^. Participants were instructed to consume the entirety of the supplement within a 5 min period. Additionally, participants were asked to replicate their diet for each of the exercise sessions and maintain normal sleep routines [21]. Participant were not aware of any experimental hypotheses or reasons for exercising at different times of day. Supplements were distributed by an independent researcher not involved in data collection, and distribution order was only divulged to researchers at the completion of all data analyses. All participants were asked to abstain from any beet or beet-derivative products during their involvement in the study. 

### 2.4. Protocol

Upon arrival to the laboratory, participants were fitted with a heart rate monitor (Polar H10, Polar Electro, Bethpage, NY, USA). Following this, participants completed a 3 min warm-up on a cycle ergometer (Monark, Varberg, Sweden) at 50 watts. Pedaling rate was standardized to 60 bpm on a metronome. Next, participants completed 3 × 15 second supramaximal WAnTs [30]. All tests were completed on an electronically braker cycle ergometer (Velotron, Racermate Inc., Seattle, WA, USA). Seat height was adjusted, recorded for subsequent visits, and situated to where the knee had approximately 5 degrees of flexion with the crank at the bottom and the foot secured to the pedal with toe straps [31]. To begin the test, participants pedaled slowly for 20 s at unloaded resistance. A 10 second countdown phase was given to allow for participants to reach maximal pedaling rate. After the countdown phase had elapsed, resistance was immediately added at a load of 7.5% of the participant’s body mass. Participants pedaled as fast and as hard as possible following the addition of resistance for a total of 15 s. The test was repeated 2 additional times with 2 min of active recovery separating WAnTs [32]. After each WAnT completion, rate of perceived exertion (RPE) was measured on a 1–10 scale where 1 was “extremely easy” and 10 was “completely exhausted” [32]. All anaerobic performance measures were calculated over each 15 s WAnT period via Velotron Software (v4 1.0.6 Velotron, Racermate Inc., Seattle, WA, USA). Specifically, for fatigue index, the following calculation was used: ((W_peak_ − W_minimum_)/15 s WAnT duration).

### 2.5. Data Analysis

All data were analyzed using Jamovi software (Version 0.9, Jamovi, Sydney, Australia). Test-to-test comparisons were drawn for anaerobic performance outcomes, heart rate, and rate perceived exertion (RPE) between each of the three conditions. Additionally, average performance over all WAnTs was also analyzed. To compare test-to-test performance, a 3 × 3 (Condition × Test) repeated measures ANOVA was used. For average performance over all three WAnTs, a 1 × 3 (Test average × Condition) repeated measures ANOVA was used. A Tukey post hoc analysis was used to detect differences in pairwise comparisons. Estimates of effect sizes for main effects were calculated using eta-squared (η^2^). All data are presented as mean ± standard deviation (SD). Significance was set at *p* ≤ 0.05 a priori.

## 3. Results

### 3.1. Anaerobic Performance

**Figure 1:**
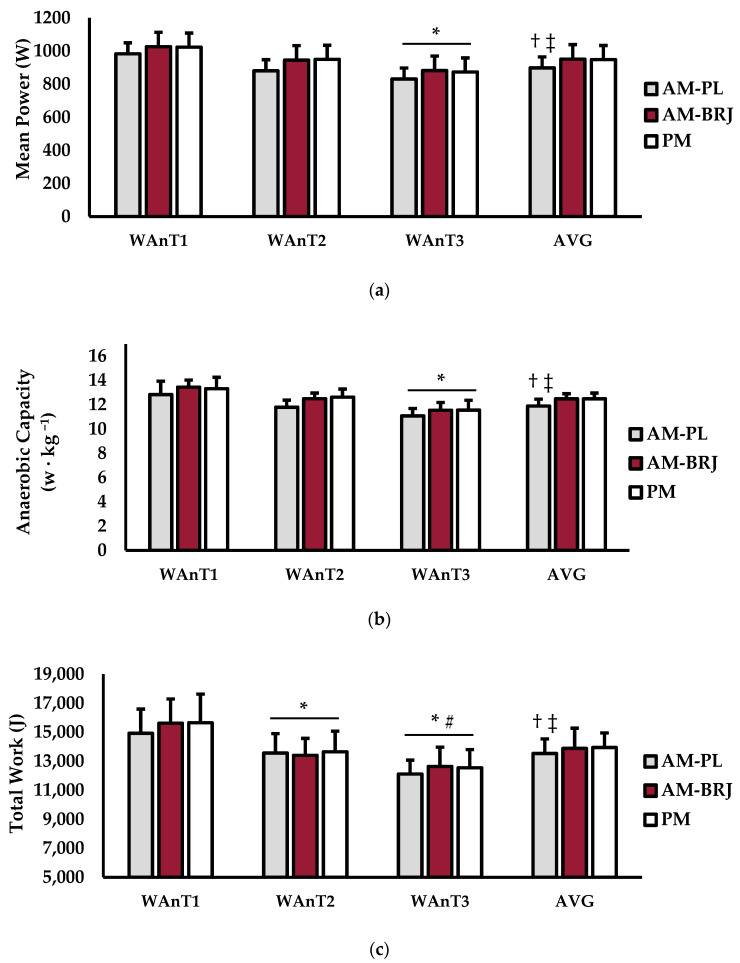
Anaerobic performance measures for Wingate 1 (WAnT1), Wingate 2 (WAnT2), Wingate 3 (WAnT3), and the average over all three WAnTs (AVG). (**a**) Mean power (watts); (**b**) anaerobic capacity (watts kilogram ^−1^); (**c**) total work (joules) over the duration of the test. Data are presented as mean ± SD; * indicates significantly different from WAnT 1 (*p* ≤ 0.05); # indicates significantly different from WAnT 2 (*p* ≤ 0.05); † indicates significantly different from PM (*p* ≤ 0.05); ‡ indicates significantly different from AM-BRJ (*p* ≤ 0.05).

Test-to-test (WAnT1, WAnT2, WAnT3) anaerobic performance variables can be seen in Figure 1. For mean power (W) (Figure 1a), there was no interaction between test × condition (*p* = 0.947; η^2^ = 0.001). There was a significant main effect for test (*p* = 0.0.26; η^2^ = 0.225) and condition (*p* = 0.002; η^2^ = 0.127). Power output during WAnT3 was significantly lower than WAnT1 (*p* = 0.007). Mean power during the AM-PL was lower than AM-BRJ (*p* = 0.035) and PM (*p* = 0.043). For anaerobic capacity (W kg ^−1^) (Figure 1b), there was no interaction between test × condition (*p* = 0.833; η^2^ = 0.003). A main effect for test (*p* < 0.001; η^2^ = 0.391) and condition (*p* = 0.008; η^2^ = 0.251) existed. Specifically, anaerobic capacity for WAnT3 was significantly lower than WAnT1 (*p* < 0.001), as well as lower in the AM-PL condition versus AM-BRJ (*p* = 0.011) and PM (*p* = 0.023). For total work (joules) (Figure 1c), there was no interaction between test × condition (*p* = 0.709; η^2^ = 0.008). There was a significant main effect for test (*p* < 0.001; η^2^ = 0.385) and condition (*p* = 0.035; η^2^ = 0.089). Total work for the AM-PL condition was lower versus PM (*p* = 0.026) and AM-BRJ (*p* = 0.022). WAnT1 was significantly higher than WAnT2 (*p* < 0.001) and WAnT3 (*p* < 0.001). Furthermore, total work for WAnT3 was significantly lower than WAnT2 (*p* = 0.047).

Average anaerobic performance variables (AVG) over all three WAnTs can also be seen in Figure 1. For mean power, there was a main effect for condition (*p* = 0.002; η^2^ = 0.127) (Figure 1a). AM-PL power output was significantly lower than PM (*p* = 0.043) and AM-BRJ (*p* = 0.035). No differences existed between PM and AM-BRJ conditions (*p* = 0.994). For anaerobic capacity, there was a main effect for condition (*p* = 0.008; η^2^ = 0.251) (Figure 1b). Anaerobic capacity was significantly lower in the AM-PL condition versus PM (*p* = 0.023) and AM-BRJ (*p* = 0.011). There were no differences between AM-BRJ and PM conditions (*p* = 0.941). A main effect for total work was observed (*p* = 0.035; η^2^ = 0.089) (Figure 1c). Total work was significantly lower in the PL-AM condition versus PM (*p* = 0.026) and AM-BRJ (*p* = 0.022). There were no differences between AM-BRJ and PM conditions (*p* = 0.933).

### 3.2. Heart Rate (HR) and Rate of Perceived Exertion (RPE)

**Figure 2:**
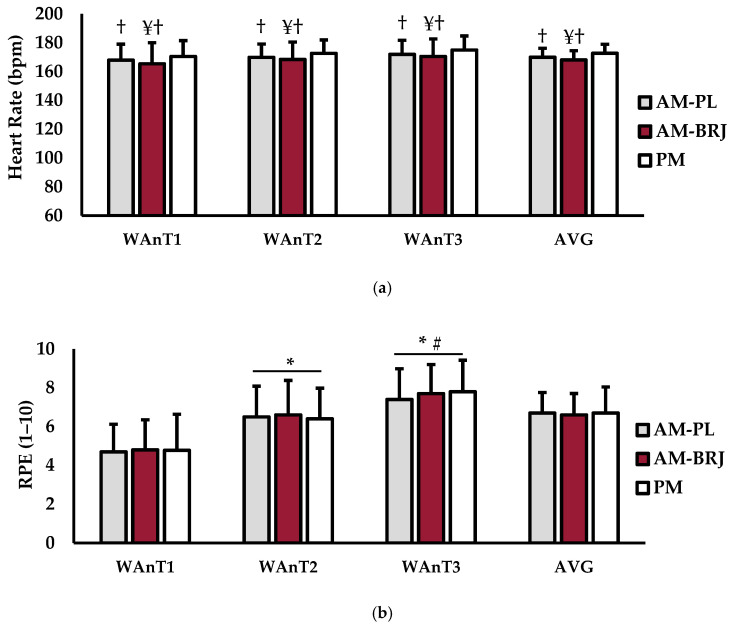
Heart rate (HR) and rate of perceived exertion (RPE) measures for Wingate 1 (WAnT1), Wingate 2 (WAnT2), Wingate 3 (WAnT3), and the average over all three WAnTs (AVG). (**a**) HR (bpm); (**b**) RPE (1–10 scale). Data are presented as mean ± SD; * indicates significantly different from WAnT 1 (*p* ≤ 0.05); # indicates significantly different from WAnT 2 (*p* ≤ 0.05); † indicates significantly different from PM (*p* ≤ 0.05); ¥ indicates significantly different from AM-PL (*p* ≤ 0.05).

Test-to-test (WAnT1, WAnT2, WAnT3) HR and RPE are shown in Figure 2. For HR (bpm) (Figure 2a), there was no main effect for test (*p* = 0.656; η^2^ = 0.028) or interaction for test × condition (*p* = 0.981; η^2^ < 0.001). However, a main effect for condition was observed (*p* < 0.001; η^2^ = 0.030). Both AM-PL (*p* = 0.002) and AM-BRJ (*p* < 0.001) HR values were significantly lower than PM. Furthermore, HR during the AM-BRJ condition was significantly lower than the AM-PL condition (*p* = 0.030). There was no main effect for RPE for condition (*p* = 0.538; η^2^ = 0.001) or interaction for test × condition (*p* = 0.780; η^2^ = 0.002) (Figure 2b). However, there was a main effect for test (*p* = 0.002; η^2^ = 0.366). RPE was significantly lower during WAnT1 than for WAnT2 (*p* = 0.002) and WAnT3 (*p* < 0.001). No differences in RPE existed between WAnT2 and WAnT3 (*p* = 0.114).

Average (AVG) HR and RPE over all three WAnTs can also be seen in Figure 2. There was a main effect for condition (*p* < 0.001; η^2^ = 0.030) for HR (Figure 2a). In particular, both AM-PL (*p* = 0.002) and (*p* < 0.001) average HR values were significantly lower than PM. Furthermore, average HR during the AM-BRJ condition was significantly lower than the AM-PL condition (*p* = 0.030). For average RPE (Figure 2b), there was no main effect for condition (*p* = 0.516; η^2^ = 0.001).

## 4. Discussion

Recent evidence has accumulated showing that NO_3_^−^ supplementation via BRJ may be beneficial to explosive exercise performance [21,22,23], but it is unknown if it can combat performance decrements in the AM. Thus, the purpose of the present investigation was to elucidate if acute BRJ ingestion could attenuate decrements of supramaximal exercise performance in the AM versus PM. Findings reveal that power output, anaerobic capacity, and total work suffer during AM-PL versus PM conditions. However, BRJ ingestion prior to supramaximal exercise in the morning (AM-BRJ) attenuated performance losses associated with the AM and restored performance to similar levels as PM. Furthermore, HR was lower during the AM trials compared to PM but BRJ potentiated HR decreases further, but no difference in RPE existed. Physiological mechanisms for which BRJ alters diurnal fluctuations in performance remain largely unclear. However, these findings have important implications for optimizing training and attenuating decreases in exercise performance during the AM. 

Currently, average power output, anaerobic capacity, and total work were lower during the AM-PL condition versus PM. This is well supported by previous investigations on time of day and WAnT performance [33,34]. Peak power during WAnTs has been reported to be lower during AM times with acrophases occurring during PM times [34]. Furthermore, Souissi et al. reported lower mean power and total work during a 30 s supramaximal WAnT in the AM versus PM, which further bolsters AM-associated decrements in performance [33]. Often, these changes in performance occur concomitantly with fluctuating body temperature. Body temperature has been shown to follow circadian patterns with temperatures being at trough levels in the morning [35]. In relation to exercise performance, increasing body and muscle temperatures lead to improved ATP-PC and glycolytic ATP turnover, muscle activation, and greater muscle force output [36,37]. While speculative, improvements in anaerobic performance with BRJ ingestion may be due to increases in body temperature, thereby leading to increased muscular force. Kuennen et al. reported that short-term BRJ supplementation led to increased core body temperature during rucksack marches in soldiers which was unexpectedly paired with improved metabolic efficiency [38]. Present improvements in performance with BRJ supplementation may be due to increases in body temperature and contractile efficiency. Supporting this, BRJ has been previously shown to reduce ATP cost of muscle force production and preserves muscle ATP-PC levels [24]. Thus, these metabolic benefits that have been previously observed with BRJ supplementation may have been responsible for attenuating decrements in performance. Previous investigations have also suggested diurnal fluctuations in intramuscular calcium handling [36,37,39]. Specifically, it has been postulated that sarcoplasmic reticulum calcium release and calcium sensitivity may be lower during AM times contributing to diurnal decreases in muscle force output [39]. Our findings of decreased power output and anaerobic capacity during the AM-PL condition versus PM support this. Decreases in calcium release and reuptake in muscle have been associated with impaired exercise performance [40]. However, dietary NO_3_^−^ has been shown to improve calcium handling and sensitivity [41,42]. Hernandez et al. showed that NO_3_^−^ enrichment resulted in higher muscle force output with increased cytosolic calcium concentrations and improved calcium handling in mice [42]. These effects were most pronounced in type II fast twitch fibers that are important for explosive exercise performance. Current data of BRJ-mediated attenuation of AM losses in anaerobic performance bolster these findings. Taken together, improvements in performance during the AM with BRJ ingestion may be due to alterations of muscle calcium handling in fast twitch fibers, thus negating AM decreases in calcium sensitivity. However, it should be cautioned that the area of time-of-day changes in skeletal muscle calcium release, especially in the context of NO_3_^−^ supplementation, remains largely understudied. The current study design alone cannot be conclusive in this regard and future studies of how BRJ influences possible diurnal changes in muscle calcium handling are a dire need in clarifying physiological mechanisms for performance enhancement. Practically speaking, these physiologic alterations may prevent decreases of muscle force output and contractile velocity during AM times. Athletic competitions often occur at varying times of day, especially in sprinters and track and field athletes, which may influence performance [43]. Current data suggest that acute BRJ intake may aid athletes in preventing performance loss during AM times which may benefit competition. Furthermore, previous evidence has shown that training at higher contractile velocities is beneficial for sprint performance and muscular adaptation while lower velocities may be less efficacious [44,45]. By eliminating power output loss during AM times with BRJ, this may allow athletes to train at higher velocities and power in the AM, ultimately leading to superior adaptations if BRJ is used chronically. However, the reader is cautioned that it is still unclear how chronic BRJ supplementation influences long-term adaptations, necessitating more investigation. 

Circadian rhythms of heart rate (HR) have been well described in a variety of populations [46,47]. In general, trough HR levels appear before and within 1–2 h of waking while peak levels appear in mid-late afternoon times [46]. While diurnal fluctuations in HR are dependent on numerous psychological and physiological factors, increases in HR during PM times may be due to increased body temperature, dietary habits, limbic system activation, and higher sympathetic neural output [48,49,50]. Present findings showed higher HR during PM conditions versus AM. Although only a small magnitude of change was observed, higher HR during PM times is in accordance with previous data [46]. However, it is not fully known if lower HR during the AM-PL condition is due to chronobiological factors or lower quantities of work performed during the AM-PL condition. Intriguingly, HR during the AM-BRJ condition was significantly lower than both PM and AM-PL despite similar or superior anaerobic capacity and total work. Although physiological mechanisms were not able to be elucidated with the current study design, lower HR with BRJ supplementation may be due to alterations in sympathetic activity and skeletal muscle blood flow [51]. Notay et al. demonstrated that acute BRJ supplementation decreases sympathetic nerve outflow and heart rate both at rest and during exercise [51]. Importantly, this investigation used the same BRJ dosing strategy as the current study. While BRJ may cause central sympathoinhibition [52], evidence has also suggested peripheral effects with lower muscle sympathetic nerve activity (MSNA) during exercise [51]. Decreases in MSNA suggest greater vasodilation and skeletal muscle blood flow. Thomas et al. showed that nitric oxide inhibits sympathetic vasoconstrictor responses to muscle during exercise thus resulting in vasodilation [53]. NO_3_^−^ supplementation through BRJ has been shown to increase local blood flow at the active muscle, preferentially to fast twitch fibers [19]. In addition to this, short-term BRJ supplementation reduces oxygen cost of contraction [24]. Thus, lower HR with BRJ may be due to increases in skeletal muscle blood flow paired with reduced oxygen cost, effectively reducing cardiovascular demand during exercise. Increases in vasodilation are important in regulating cardiac output, which is largely dependent on oxygen delivery [54]. Further study on what mechanisms control HR with BRJ supplementation and how this may be different depending on the time of day are warranted. Lastly, RPE remained unchanged between conditions. Lack of changes in RPE between AM and PM times have been reported previously in explosive resistance exercise [6]. Recently, Jorda et al. reported no changes in general RPE with BRJ supplementation prior to a 30 s WAnT [25]. However, muscle RPE was significantly lower with BRJ supplementation, indicating that changes in perceived exertion may be localized to the working muscle rather than overall perception. BRJ ingestion has been previously linked to increased lactate clearance that could be a contributing factor to localized RPE reductions [20]. Overall, BRJ supplementation may be useful for altering physical fatigue in athletes but may not be applicable to those seeking to decrease perceptive fatigue. Future investigations are required to delineate physiological and subjective changes with BRJ ingestion at varying times of day.

## 5. Conclusions

While current findings provide novel insight into how BRJ alters diurnal fluctuations in exercise performance, there were several limitations. First, blood NO_2_^−^ was not directly measured. While other investigations have used the same approach [21], it is unknown what plasma concentration of NO_2_^−^ is necessary for attenuation of diurnal losses in performance. However, it should be mentioned that this exact same dosing strategy has been repeatedly shown to result in increased plasma NO_2_^−^ [28,55]. Most of the previously discussed physiological mechanisms for improvements in AM performance with BRJ ingestion remain largely unclear. To identify specific mechanisms responsible for changes in performance, a more in-depth study measuring neuromuscular and metabolic changes with AM BRJ supplementation is warranted. Only trained male sprinters were studied currently. Previous evidence has suggested NO_3_^−^ metabolism may be different in females and other populations [56]. Thus, current findings may not be generalizable to other populations or athletes. Lastly, the current study design did not include a BRJ-PM group, leaving it unclear to what degree BRJ might improve performance in trained sprinters during afternoon times and if magnitudes of change are similar between AM and PM. However, the central aim of this investigation was to identify if BRJ could specifically prevent AM-associated losses in power output, and there is a multitude of investigations showing similar magnitudes in performance increases when time of day is standardized [21,22,23,25]. This same study design approach has been used successfully in previous work on caffeine supplementation and diurnal fluctuations in power output [10]. Lastly, sleep and overall dietary intake were not strictly controlled for, although participants were encouraged to maintain similar routines throughout participation. We cannot rule out the possibility of these factors influencing the results to a degree. 

In conclusion, supramaximal exercise performance suffers during AM versus PM times of day in trained male sprinters. However, acute BRJ ingestion attenuates decreases in AM exercise performance and results in similar performance levels to PM times. While RPE remained unchanged, BRJ ingestion resulted in lower HR compared to PM and AM-PL conditions despite similar or higher total work and power output. Athletes and coaches looking to optimize training during AM times may utilize BRJ in efforts to prevent worsening performance. Future investigation on whether performance changes affect adaptations chronically are needed and may provide a safe and effective dietary intervention to improve AM training. 

## Data Availability

The data for the current study is available within this manuscript.
